# Thyroid hormones, mitochondria, aging, and cancer

**DOI:** 10.3389/fendo.2025.1682089

**Published:** 2026-01-05

**Authors:** Gennadi Glinsky, Aleck Hercbergs, Paul J. Davis

**Affiliations:** 1 Institute of Engineering in Medicine, University of California San Diego, San Diego, CA, United States; 2 Cleveland Clinic, Cleveland, OH, United States; 3 Department of Medicine, Albany Medical College, Albany, NY, United States

**Keywords:** Thyroid hormone (t3-t4), tetrac, mitochondria, aging, cancer

## Abstract

In mammals, thyroid hormones (THs) and their metabolites exert their regulatory effects on metabolism, oxygen consumption, energy generation and expenditure, and gene expression via genomic and non-genomic mechanisms by binding to different types of THs receptors. THs binding to nuclear receptors engages the genomic pathway, while binding to receptors on mitochondria or cell membrane receptors located on thyrointegrins initiates nongenomic mode of actions. In this contribution, we review the effects of THs and their metabolites on mitochondrial structure and functions, including both classical and non-canonical features, in various pathophysiological conditions, focusing on aging, stemness, and cancer. Our analysis indicates that nongenomic mode of actions of THs on mitochondria appear affected during aging and its alterations may contributed to development of cancer and other aging-associated disorders.

## Introduction

Thyroid hormones (THs), primarily triiodothyronine (T3) and thyroxine (T4), are crucial regulators of metabolic rate, oxygen consumption, and energy expenditure in mammals. T3 is widely regarded as the only form of thyroid hormone that has a substantive role to play in the control of metabolism in normal and malignant cells. Thyroid hormones exert their pleiotropic effects on biological functions via the genomic and non-genomic mechanisms by binding to different types of thyroid hormone receptors (THRs): i) TH nuclear receptors located in cytosols and translocated to nucleus upon T3 binding to affect gene expression (genomic pathway); ii) TH receptors located on mitochondria or on the cell surface membrane (non-genomic pathway) (1, 2).

Thyroid hormone is a critical contributor to regulation of cellular development and control of energy expenditure (metabolism). (1, 3). The principal thyroid hormone product of the thyroid gland is L-thyroxine (T4), conventionally viewed as a prohormone source via deiodination of 3,5,3’-tri-iodo-L-thyronine (T3) (3). T3 is the primary ligand in normal cells of nucleoprotein thyroid hormone receptors (TRs) and of an energy-regulating mitochondrial receptor (3). TRs are the basis for what are designated genomic actions of thyroid hormone in normal cells. Detailed analyses of the main forms of thyroid hormones and their isoforms, their primary target organs, and ultimately their connection to mitochondria has been reported elsewhere (4). In cancer cells, however, T4 serves as a principal thyroid hormone, acting nongenomically via a membrane-located receptor on integrin alphavbeta3 (1, 3). From this receptor, physiological concentrations of T4 can regulate cell division, metastasis and protective radioresistance and chemoresistance (1) One of the focus of the present analysis was to examine the evidence for actions of T4 via the thyrointegrin in cancer cells on mitochondria and energy-regulation.

Integrins are ubiquitous cell surface receptor molecules that function as crucial mediators of cellular adhesion to extracellular matrix, leucocytes adhesion to endothelium, as well as cell-surface receptors for various growth factors, small molecules, and hormones, including thyroid hormones and their metabolites (reviewed in 5). The relatively high expression of the αVβ3 intergrin (designated as thyrointegrin) on cancer cells and on dividing endothelial cells apparently enables T4 to influence malignant and endothelial cells’ growth and metabolism (1, 6). The thyrointegrin expression permits certain endogenous T4 metabolites, such as tetrac, to disrupt cancer cell metabolism, without significantly affecting normal, nonmalignant cells in which the thyrointegrin is not active, non-accessible, or is present only in minute quantities. Human cancer cells contain an activated form of thyrointegrain receptor that binds L-thyroxine (tetraiodothyronine) and tetrac (tetraiodothyroacetic acid) and signaling via thyrointegrin may permit to control a number of mitochondrial functions. Thus, with respect to normal cells, T4 serves as a prohormone for T3, but during carcinogenesis T4 broadly functions with respect to cancer cells as a hormone and these T4 activities likely include regulation of mitochondrial function, stemness phenotype, and apoptosis (7, 8). Notably, tetrac (tetraiodothyroacetic acid) functions as a potent inhibitor of T4 activities and exerts marked antitumor effects in experimental models of human cancers. Another metabolite of THs, reverse T3 (rT3) appears to function via interactions with αvβ3 integrin receptors and nongenomically activating signaling pathways that restored gamma-glutamate transferase activity (9). The crystal structure of the complex of human transthyretin (hTTR) with 3,3’,5,5’-tetraiodothyroaceticacid (T4Ac) has been determined to 2.2 Angstrom resolution (10). Molecular modeling and combined quantum mechanical and molecular mechanical (QM/MM) methods demonstrate that 3,5’-triiodothyronine (T(3)) and 3,5,3’,5’-tetraiodothyroacetic acid (T(4)ac) bound in two different modes, occupying two alternate sites, one of which is along the Arg side chain of the RGD cyclic peptide site on the αVβ3 intergrin molecule (11, 12). Collectively, these observations facilitated the development of a novel family of anticancer agents based on tetrac molecules, among which one of the leading drug candidates is currently undergoing the FDA-approved Phase I clinical trials. Nano-formulated tetrac (Nanotetrac) exerts profound effects on stemness and energy metabolism gene expression programs (8). These findings suggest that certain mitochondrial functions may serve as the promising molecular targets for anticancer drug development and molecular interference with mitochondrial functions is likely mediating, in part, therapeutic effect of the Nanotetrac on malignant growth. These observations also implied the mechanistic links at the gene expression crosstalk level between the stemness and the energy metabolism gene expression programs, thus suggesting the link between mitochondria functions and the stemness phenotype (8).

The widespread physiological actions of thyroid hormones are linked to their marked influence on mitochondria, the cellular energy-generation powerhouses responsible for ATP production through oxidative phosphorylation. Next we summarize evidence of the diverse effects of thyroid hormones on mitochondria, including effects on mitochondrial biogenesis, respiration, uncoupling, mitochondrial dynamics, and reactive oxygen species (ROS) production.

In summary, there are multiple lines of evidence for two key mechanisms of Thyroid Hormone actions (**Table 1**), collectively indicating that Thyroid hormones (THs), primarily triiodothyronine (T3) and thyroxine (T4), regulate metabolism and gene expression through two main pathways:

**Table 1:** Mechanisms of thyroid hormone (TH) action.

Mechanism	Receptor location	Primary TH agent	Key functional effect	Implications in cancer
Genomic	Nucleus	**T3** (Triiodothyronine)	Regulates long-term gene expression (e.g., mitochondrial biogenesis, metabolic enzymes).	Essential for normal cell function; dysregulation affects growth/differentiation.
Non-Genomic (Membrane)	Cell surface (Integrin αVβ3)	**T4** (Thyroxine)	Activates MAPK pathway; promotes angiogenesis, proliferation, and anti-apoptosis.	Critical: T4 acts as a pro-malignancy hormone via this receptor in cancer cells.
Non-Genomic (Mitochondrial)	Inner Mitochondrial Membrane (IMM)	**T3** and potentially metabolites	Rapid, acute stimulation of respiration, oxygen consumption, and mitochondrial integrity.	Directly influences cancer cell bioenergetics and survival.

1. Genomic Pathway: T3 binds to nuclear receptors (THRs) to affect gene expression.
2. Non-Genomic Pathway: THs bind to receptors located directly on mitochondria or on cell-surface thyrointegrins (specifically the αVβ3 integrin) to initiate rapid actions.

Principal distinctions of these mechanisms are illustrated by the relationships of T4 and the Thyrointegrin in physiological conditions and cancer. In normal cells, T4 primarily serves as a prohormone for the active T3. In cancer cells, T4 functions as a hormone by binding to the activated αVβ3 integrin (thyrointegrin), which influences malignant cell growth, metabolism, stemness, and apoptosis. The TH metabolite tetrac (tetraiodothyroacetic acid) acts as a potent inhibitor of T4’s malignancy-promoting activities via the thyrointegrin, and its nano-formulated version, Nanotetrac, is being developed as an anticancer agent.

## Effects of thyroid hormones on mitochondrial biogenesis

Thyroid hormones are well-established as major regulators of mitochondrial biogenesis, the process of forming new mitochondria. T3, the biologically active form, exerts this effect largely through genomic mechanisms, binding to thyroid hormone receptors (TRs) in the nucleus. This binding modulates the expression of genes involved in mitochondrial function and biogenesis. Key targets include nuclear respiratory factor 1 (NRF1) and peroxisome proliferator-activated receptor gamma coactivator 1 alpha (PGC-1α), both of which are master regulators of mitochondrial biogenesis. Upregulation of these factors leads to increased synthesis of mitochondrial proteins and components of the respiratory chain (13, 14). Beyond genomic actions, there is evidence for direct, non-genomic effects of T3 on mitochondrial biogenesis. These rapid effects can occur independently of nuclear gene expression and involve direct interactions with mitochondrial components (14).

## Effects of thyroid hormones on mitochondrial respiration and oxidative phosphorylation

THs significantly impact mitochondrial respiration and the efficiency of oxidative phosphorylation (OXPHOS). Hyperthyroidism generally leads to an increase in oxygen consumption and an accelerated metabolic rate, while hypothyroidism results in the opposite. This calorigenic effect is mediated by alterations in the expression of respiratory genes, encoded both nuclear and mitochondrial DNA, ensuring the stoichiometric assembly of the respiratory chain (15, 16). Specifically, thyroid hormones enhance the activity of various components of the electron transport chain, increasing the rates of oxidation of substrates such as succinate, glutamate, and beta-hydroxybutyrate. They also influence the activity of membrane-bound enzymes like alpha-glycerophosphate dehydrogenase (15). This intricate regulation optimizes the cell’s ability to generate ATP in response to metabolic demands.

## Effects of thyroid hormones on mitochondrial uncoupling and thermogenesis

A crucial aspect of TH action on mitochondria is their role in regulating thermogenesis through mitochondrial uncoupling. The concept of “uncoupling” refers to the dissipation of the proton gradient across the inner mitochondrial membrane, diverting energy from ATP synthesis towards heat production. Uncoupling proteins (UCPs), particularly UCP1 (primarily in brown adipose tissue) and UCP3 (abundant in skeletal muscle), are key mediators of this process (17–19).

Thyroid hormones, especially T3, are potent inducers of UCP gene expression. For instance, T3 directly increases UCP3 mRNA and protein levels, leading to a decrease in mitochondrial respiratory efficiency and an increase in resting metabolic rate. This provides a direct link between T3 and thermogenesis (17). Furthermore, the less active thyroid hormone metabolite, 3,5-diiodothyronine (3,5-T2), has also been shown to rapidly stimulate mitochondrial uncoupling and increase oxygen consumption, potentially through direct effects on mitochondrial components (20).

## Effects of thyroid hormones on mitochondrial dynamics and quality control

Beyond biogenesis and respiration, THs are increasingly recognized for their influence on mitochondrial dynamics, including fusion, fission, and mitophagy. These processes are essential for maintaining a healthy mitochondrial network and adapting to changing cellular energy demands. While research in this specific area is evolving, studies suggest that THs can coordinate signals from both nuclear and mitochondrial genomes to regulate mitochondrial quality control. For example, T3 has been shown to stimulate autophagy, a process crucial for the removal of damaged mitochondria (mitophagy), which is essential for subsequent mitochondrial biogenesis and activity in skeletal muscle (21). Disruptions in TH signaling can lead to mitochondrial dysfunction and contribute to metabolic disorders.

## Effects of thyroid hormones on reactive oxygen species production

Mitochondria are a primary source of reactive oxygen species (ROS) as a byproduct of oxidative phosphorylation. The relationship between THs and mitochondrial ROS production is complex and can be context-dependent. While increased metabolic activity induced by THs can lead to elevated ROS generation, THs also play a role in regulating antioxidant defense mechanisms (22).

Some studies suggest that hyperthyroidism can increase free radical production and lipid peroxide levels due to heightened metabolic rates. Conversely, hypothyroidism may lead to decreased ROS production due to metabolic suppression (22). However, THs also appear to have protective effects against mitochondrial oxidative stress, potentially by increasing the activity and expression of uncoupling proteins and mitoKATP channels, which can help to reduce ROS formation (23). The precise balance between ROS generation and antioxidant defense mediated by THs is crucial for maintaining cellular homeostasisc.

In summary, large body of evidence document regulatory actions of THs on Classical mitochondrial functions (**Table 2**).

**Table 2:** Thyroid hormone influence on mitochondrial functions.

Mitochondrial process	Action of TH (T3/T4)	Functional outcome	Link to pathophysiology
Biogenesis	**T3** upregulates key transcription factors (e.g., PGC-1α, NRF1).	Increases mitochondrial mass and content.	Essential for tissue maintenance and recovery; declines in aging.
Respiration/OXPHOS	**T3** rapidly stimulates complex activity.	Increases basal metabolic rate and oxygen consumption (calorigenic effect).	Directly linked to hyper/hypothyroidism symptoms.
Uncoupling (UCPs)	**T3** induces uncoupling proteins (UCPs).	Dissipates proton gradient to generate heat instead of ATP.	Thermogenesis; may reduce damaging Reactive Oxygen Species (ROS).
Quality Control	**T3** stimulates mitophagy and mitochondrial turnover.	Eliminates damaged mitochondria, maintaining cell health.	Impaired mitophagy is a hallmark of aging and contributes to neurodegeneration.

THs are essential regulators of mitochondrial activity, mediating both immediate and long-term effects.

In Mitochondrial Biogenesis (Creation of New Mitochondria), T3 represents a major regulator, primarily via the genomic pathway, by upregulating master regulators like NRF1 and PGC-1α.

In Respiration & OXPHOS, THs actions are illustrated by hyperthyroidism, which increases oxygen consumption and metabolic rate (calorigenic effect) by enhancing the activity of the electron transport chain components.

In the processes of Uncoupling & Thermogenesis, T3 induces uncoupling proteins (UCPs, like UCP3) to dissipate the proton gradient for heat production instead of ATP synthesis, directly linking T3 to thermogenesis.

In mitochondrial Quality Control, T3 has been shown to stimulate autophagy (specifically mitophagy, the process of the removal of damaged mitochondria), which is crucial for mitochondrial turnover and biogenesis.

In reactive oxygen species (ROS) Production THs play a crucial regulatory role: while increased metabolism from THs can facilitate an increase of ROS levels, THs also have a protective role by regulating antioxidant defenses and increasing UCPs to reduce ROS formation.

## Rapid non-genomic effects of thyroid hormones mediated by thyroid hormone receptors on mitochondria

Thyroid hormones (THs), primarily triiodothyronine (T3), are crucial regulators of metabolism, growth, and development. While their most well-established mode of action involves binding to nuclear thyroid hormone receptors (THRs) to regulate transcription and gene expression, a growing body of scientific evidence points to the presence and functional significance of THRs directly on mitochondria, mediating rapid, non-genomic effects of THs.

### Discovery and initial characterization of thyroid hormone receptors on mitochondria

The concept of direct mitochondrial TH actions emerged decades ago with observations of rapid thyroid hormone effects on mitochondrial oxygen consumption that were too fast to be explained by genomic mechanisms mediated by nuclear THRs. Sterling et al. (2) provided early evidence of high-affinity, low-capacity binding sites for thyroid hormone on submitochondrial fractions, specifically localizing them to the inner mitochondrial membrane (2, 24). This seminal work suggested the presence of a structurally and functionally distinct mitochondrial thyroid hormone receptors on mitochondria.

### Identification of specific thyroid hormone receptor isoforms

Subsequent research identified specific THR isoforms within the mitochondria and investigated their contributions to mitochondrial function. *Shortened THR isoforms:* Studies have indicated that N-terminus shortened thyroid hormone receptor (sTHR) isoforms, derived from alternative translation initiation sites of the TRα gene, are present in mitochondria (25). These sTHRs have been shown to acutely influence mitochondrial metabolism, acutely increased mitochondrial membrane potential and oxygen consumption upon T3 stimulation. Analysis of thyroid hormone receptors localization in target cells and tissues revealed the complex interplay of TR’s dynamic transport pathways, including targeting to the nucleus, cytoplasm, and mitochondria (26).

### Functional roles of mitochondrial THRs

The presence of THRs on mitochondria implies direct effects on mitochondrial processes, independent of effects on nuclear gene expression. These non-genomic effects of mitochondrial THRs are crucial for rapid metabolic adjustments.

#### Direct modulation of mitochondrial metabolism

Mitochondrial THRs are involved in the acute stimulation of mitochondrial oxygen consumption and energy production. This can involve switching metabolism towards more efficient oxidative phosphorylation (25).

#### Regulation of mitochondrial biogenesis and function

Thyroid hormones, through both genomic and non-genomic pathways, profoundly influence mitochondrial biogenesis (the formation of new mitochondria) and overall mitochondrial activity, in particular, influencing the mitochondrial protein import by upregulating components of protein import machinery (21, 27). While nuclear TRs play a role in regulating genes involved in mitochondrial biogenesis (e.g., PGC-1α), direct mitochondrial TRs can contribute to the rapid functional changes such as stimulation of autophagy, which is crucial for mitochondrial turnover and biogenesis (21).

#### Anti-apoptotic effects

Some studies have shown that T3-activated sTRs localized to mitochondria can mediate anti-apoptotic effects, independent of nuclear transcriptional activity (25). Furthermore, it is important to underscore that in the cellular context of mitochondrial DNA damage T3 actions affect the mDNA repair. Specifically, both T2 and T3 have been shown to facilitate the repair of induced mitochondrial DNA lesions (28).

The growing body of scientific evidence unequivocally demonstrates the presence of thyroid hormone receptors on mitochondria, including specific shortened isoforms of TRα. These receptors mediate rapid, non-genomic effects of thyroid hormones on mitochondria. Mitochondrial THRs play critical roles in rapid, acute regulation of mitochondrial metabolism, oxygen consumption, and potentially contribute to mitochondrial biogenesis and activation of anti-apoptotic pathways. Direct mitochondrial pathway of THs actions adds a significant layer of complexity and fine-tuning to the overall physiological actions of thyroid hormones, complementing their well-understood genomic effects mediated by binding to nuclear THRs. Further research should continue to elucidate the precise mechanisms and physiological implications of this important non-genomic signaling pathway.

Therefore, Rapid Non-Genomic Actions on Mitochondria represent an important pathway of THs regulatory actions. A distinct set of Thyroid Hormone Receptors (THRs), including shortened TRα isoforms (sTHRs), are localized on the inner mitochondrial membrane. These receptors mediate rapid, non-genomic effects of T3, such as the acute stimulation of oxygen consumption, and may also contribute to anti-apoptotic effects.

### Non-canonical functions of mitochondria

Thyroid hormones profoundly impact cellular energy metabolism by exerting multifaceted regulatory control over mitochondria. Their actions range from promoting mitochondrial biogenesis and enhancing respiratory chain activity to modulating uncoupling and influencing mitochondrial dynamics and ROS production. These diverse effects, mediated by both genomic and non-genomic mechanisms, underscore the critical role of THs in maintaining metabolic health and highlight mitochondria as a central hub for thyroid hormone actions. Further research continues to unravel the intricate molecular pathways by which these essential hormones fine-tune mitochondrial physiology. Recent advances in mitochondria research identified numerous previously unknown aspects of mitochondrial structural features and biological activities. While direct experimental evidence documenting the effects of thyroid hormones on these recently discovered features of the mitochondrial lifecycle are lacking, it is highly likely that they will be affected by thyroid hormones and their metabolites. In the next sections we will highlight some of the recent advances in mitochondria research focusing on aspects that are most likely relevant to cancerogenesis and aging.

### Intercellular transfer and trafficking of mitochondria

Intercellular mitochondrial transfer is a rapidly emerging field focusing on the dynamic exchange of mitochondria between cells, describing a novel mechanism of cell-to-cell communication and cellular rescue, as well as offering exciting novel therapeutic opportunities. These processes have been observed across various cell types and pathophysiological conditions, suggesting their broadly significant physiological and pathological impacts. First step in intercellular transfer and trafficking of mitochondria is the release of mitochondria from donor cells.

Mitochondria can be released from donor cells through several mechanisms:

#### Tunneling Nanotubes (TNTs) pathway

These are F-actin-rich, membranous bridges that connect cells, forming direct conduits for the active transfer of various cellular components, including mitochondria. TNT-mediated transfer is a highly efficient and well-documented mechanism. Studies have shown that stress conditions can induce TNT formation and enhance mitochondrial transfer (29, 30).

#### Extracellular vesicles pathway

This category includes exosomes, microvesicles, and apoptotic bodies. Mitochondria, or mitochondrial components, can be packaged within these vesicles and released into the extracellular space. EVs can then be taken up by recipient cells, delivering their mitochondrial cargo. While less characterized than TNTs for intact mitochondrial transfer, EV-mediated transfer of mitochondrial DNA and proteins is recognized (31, 32).

#### Mitochondrial exocytosis pathway

This involves the direct extrusion of mitochondria from the cell surface, often observed in response to stress or damage, as a mechanism to clear dysfunctional mitochondria. This process can involve lysosomal pathways (33).

Next step in intercellular transfer and trafficking of mitochondria is the uptake of mitochondria by recipient cells. Recipient cells can internalize extracellular mitochondria through various endocytic pathways:

#### Phagocytosis pathway

Larger mitochondria or mitochondrial fragments can be engulfed by phagocytic cells (34).

#### Macropinocytosis pathway

This is a clathrin-independent endocytic pathway involving the non-specific uptake of extracellular fluid and its contents, including mitochondria (33).

#### Receptor-Mediated Endocytosis pathway

While less understood for whole mitochondria, specific surface receptors on recipient cells might recognize and internalize mitochondria, potentially through interaction with mitochondrial outer membrane proteins (35, for general EV uptake).

Perhaps, one of the most dramatic recent examples of mitochondrial transfer is neuronal transfer of mitochondria to cancer cells that appears to promote metastatic spread. Cancer cells hijack mitochondria from neuron using ultrathin nanotubes connecting two types of cells and facilitating trafficking of neuronal mitochondria from neurons to cancer cells (36). Importantly, cancer cells that accumulated neuronal mitochondria appear to acquire the propensity to spread more efficiently because lineage tracing and fate mapping experiments revealed their selective enrichment at metastatic sites following dissemination from primary tumors (36). It seems likely that accumulation of neuronal mitochondria by cancer cells endow them increased ability to survive the circulatory stress during metastatic dissemination and colonization of distant organs.

Interestingly, it has been reported that intercellular nanotubes mediate mitochondrial trafficking between cancer and immune cells whereby cancer cells hijack the mitochondria from immune cells via physical nanotubes connecting two cell types (37). The nanotube-mediated transfer of mitochondria from immune cells to malignant cells metabolically empowers the cancer cells and caused depletion of the immune cells because inhibition of the nanotube assembly machinery significantly reduced mitochondrial transfer and prevented the depletion of immune cells (37).

Remarkably, cancer cells’ defective mitochondria with mtDNA mutations are readily transferred from cancer cells to tumor-infiltrating lymphocytes, TILs (38). Physiologically mitochondria in TILs readily undergo mitophagy through mechanism driven by reactive oxygen species. In striking contrast, mitochondria containing mutant mtDNA transferred from cancer cells to TILs do not undergo mitophagy, reportedly due to mitophagy-inhibitory molecules, which are attached to mitochondria and together are transferred to TILs (38). Consequently, transfer of mitochondria containing mutant mtDNA results in homoplasmic replacement of “healthy” mitochondria in TILs. TILs that acquired cancer cells’ mitochondria with mtDNA mutations manifest metabolic abnormalities, undergo senescence with defects in effector functions and memory formation, which, in turn, leads to impaired antitumor immunity (38). These observations revealed previously unknown mechanism of cancer immune evasion through transfer of defective mitochondria from malignant cells to immune cells residing in tumor microenvironment.

Intriguingly, accumulation of mitochondria containing mutant mtDNA has been documented during mammalian aging (39). Single-cell DNA sequencing of murine and human hepatocytes revealed age-associate increases in abundance of mutant alleles in DNA sequences governing mtDNA replication. These mutated alleles appear to provide a replicative advantage for mitochondria harboring mutated mtDNA (39}. Thus, continuing accumulation of mitochondria containing mutated mtDNA would provide a mechanism driving the age-associated erosion of structural and functional integrity of mtDNA and dysfunction of mitochondria. It has been demonstrated that mtDNA mutations can be genetically corrected and normal metabolic function restored, thus causing a metabolic rescue in pluripotent stem cells from patients with mutant mtDNA disease (40). Mitochondrial dysfunctions have been linked to aging-associated chronic inflammation and cellular senescence, in particular, senescence of immune cells, as well as pathogenesis and clinical manifestation of broad spectrum of aging-associated pathological conditions, including cancer, neurodegeneration, and cardiovascular diseases (41). Given the profound multifaceted regulatory effects of thyroid hormone(s) on mitochondrial structural and functional characteristics (see above), it seems reasonable to expect that many (if not all) novel, recently discovered facets of mitochondrial roles in physiology and pathologies of human cells, tissues, and organs likewise will affected by thyroid hormone(s) and/or their metabolites. These aspects of regulatory effects of thyroid hormone(s) on mitochondria should be a subject of intense interest for both fundamental and clinical investigations.

Non-Canonical Mitochondrial Functions and Pathophysiology are rapidly expanding our understanding of human physiology and pathologies. We highlighted newly discovered mitochondrial roles that are likely affected by THs in disease and aging.

- Intercellular Mitochondrial Transfer: Mitochondria can be physically transferred between cells via mechanisms such as Tunneling Nanotubes (TNTs), which is an emerging form of cell-to-cell communication and cellular rescue.
- Role in Cancer Immune Evasion: Cancer cells have been observed to hijack healthy mitochondria from immune cells via nanotubes, metabolically empowering the cancer cell. Furthermore, cancer cells transfer defective mitochondria with mutant mtDNA to tumor-infiltrating lymphocytes (TILs), causing the TILs to become dysfunctional and contributing to cancer immune evasion.
- Mitochondria and Aging: The accumulation of mitochondria containing mutant mtDNA is observed during mammalian aging, a process that is thought to drive the age-associated erosion of mitochondrial integrity and dysfunction. Mitochondrial dysfunctions are linked to aging-associated disorders like chronic inflammation, cellular senescence, and cancer.

## Mitochondria effects on stemness phenotype

Mitochondria play a crucial role in regulating stem cell fate and maintaining the “stemness” phenotype, which encompasses self-renewal capacity and pluripotency. Stem cells, particularly pluripotent stem cells (PSCs), exhibit a unique metabolic signature characterized by a reliance on glycolysis even in the presence of oxygen, often referred to as “aerobic glycolysis” or the Warburg effect. This metabolic state is intrinsically linked to their stemness phenotype.

### Mitochondrial structural-functional dynamics and metabolism in stemness and differentiation

Metabolic Shift during differentiation of stem cells. Undifferentiated PSCs possess immature, fragmented mitochondria and rely heavily on glycolysis for ATP production. Upon differentiation, there is a metabolic switch towards oxidative phosphorylation (OXPHOS), accompanied by mitochondrial maturation, fusion, and increased biogenesis. This metabolic shift is essential for lineage specification (42–44) and it is intrinsically linked to mitochondrial morphology and functions.

Mitochondrial Morphology and Function. The highly fragmented and less active mitochondrial network in PSCs contributes to lower reactive oxygen species (ROS) production, which is crucial for maintaining genome stability by decreasing the likelihood of mutations and preventing cellular differentiation. Conversely, mitochondrial fusion and increased OXPHOS activity are associated with cellular differentiation (45).

Mitochondrial Biogenesis and Quality Control. Processes like mitochondrial biogenesis (through PGC-1α pathway) and mitophagy (selective degradation of damaged mitochondria) are tightly regulated in stem cells to maintain a healthy mitochondrial pool and ensure proper metabolic function. Aberrations in these processes can severely impair stemness phenotype (46).

### Therapeutic potential of mitochondrial transfer on stemness phenotype

The transfer of healthy mitochondria from mesenchymal stem cells (MSCs) or other supportive cells to compromised somatic cells or even other stem cells has been shown to enhance their viability, functional capacity, and in some contexts, may improve or restore aspects of the stemness phenotype in injured tissues and believed to have a significant therapeutic potential across the broad range of diseases (47).

### Effects of mitochondrial unfolded protein response pathway on stemness phenotype

It has been reported that the unfolded protein response of the endoplasmic reticulum (UPR^ER^), the mitochondrial UPR, and the heat shock response, which ensure proteome quality during stress, are transiently activated during reprogramming of somatic cells into PSCs (48). A c-Myc-dependent, transient decrease in mitochondrial proteolysis, is accompanied by mitochondrial UPR activation at the early phase of pluripotency acquisition (49). However, extended UPR activation impedes the mesenchymal-to-epithelial transition (MET) causing the inhibition of pluripotency acquisition. Mitochondrial signaling is linked to the regulation of the cells’ epigenetic state and cell fate decisions via modulation of H3K9Ac levels. Activation of c-Jun enhances the expression of acetyl-CoA metabolic enzymes and reduces acetyl-CoA levels, affecting levels of H3K9Ac. c-Jun activity decreases the occupancy of H3K9Ac at MET genes, further inhibiting MET and pluripotency acquisition (49), thus underscoring the crucial role of mitochondrial UPR-modulated MET in pluripotent stem cell plasticity. Activation of mitochondrial the UPR promotes cancer cell migration and invasion by enhancing epithelial-to-mesenchymal transition (EMT) suggesting a role of mitochondrial UPR-activated EMT in cancer metastasis.

### Essential roles of mitochondria and thyroid hormones in proper execution of stemness phenotype-related functions *in vivo* during embryogenesis, tissue regeneration, and aging

Several studies document the essential roles of mitochondria and thyroid hormones for proper functions of stem cells during embryonic development and tissue regeneration. While PSC can survive in culture without mitochondria for several days, reducing mitochondrial abundance leads to delayed development in pre-implantation mouse embryos (50). Notably, fetal neurogenesis depends on thyroxine; thyroid hormones induce transcriptional changes that promote the progression of human neural precursor cells, and optimal thyroid hormone levels is necessary for human neural precursor cells to differentiate into neurons (51). Inhibition of 2-oxoglutarate dehydrogenase (OGDH) or supplementation with α-ketoglutarate (αKG) reversed impaired differentiation and maturation of secretory cells and promoted tissue healing (52), consistent with the concept that the activities of the mitochondrial tricarboxylic acid (TCA) cycle enzymes and TCA metabolites regulate cell fate during tissue regeneration.

During aging, hematopoietic stem cells (HSCs) manifest progressively diminishing self-renewal potential and exhibit myeloid-biased differentiation concomitantly with a marked decline in their ability to facilitate normal hematopoiesis and diminished execution of adaptive immune functions. Recent experiments identified upregulation of Clusterin as a principal cause of aging-associated myeloid bias of HSCs (53). Clusterin promotes mitochondrial hyperfusion and its ablation attenuated oxidative phosphorylation, improved mitophagy, and reversed myeloid-biased differentiation via the OXPHOS-p38-Cebpb axis. Therefore, these observation established Clustering upregulation acting on Mfn2-OXPHOS-p38-Cebpb axis in mitochondria as the mechanism of impaired functions of aged HSCs leading to dominant myeloid-biased differentiation (53).

Important aspect of mitochondrial functions related to stemness and differentiation phenotypes is mitochondrial control of inflammation (54). Mitochondrial control of inflammation is executed by mitochondrial constituents and metabolic products, which when released from damaged (permeabilized) mitochondria into cytosol or extracellular space are recognized as damage-associated molecular patterns (DAMPs) and promote inflammatory responses. Failure to dispose damaged mitochondria triggers pathological inflammatory reactions associated with multiple human disorders, which is often occurs during aging (55).

Establishment and maintenance of dynamic connections between mitochondria and endoplasmic reticulum through endoplasmic reticulum-mitochondria contact sites are essential for rapid real-time inter-organelle exchange and integration of information on metabolic states and pathophysiological cues. Genetic and biochemical studies established the functional role of endoplasmic reticulum-mitochondria contact sites in key signaling processes at the cellular and organ levels, including Ca2+ fluxes, apoptosis, mitochondrial dynamics, metabolism and lipid homoeostasis (56). Most recently, endoplasmic reticulum-mitochondria contact sites have been established as the prime hotspots of the membrane phospholipids’ peroxidation driving ferroptosis (57, 58) in normal and malignant cells. The term ferroptosis defines an iron-dependent form of regulated cell death driven by lipid peroxidation, which is recognized as one of the important distinct types of programmed cell death in physiological and pathological conditions (57, 58).

## Regulatory effects of mitochondria on gene expression

Beyond their canonical role in energy production, mitochondria exert profound regulatory effects on gene expression in the nucleus, influencing diverse cellular processes including differentiation, proliferation, metabolism, and stress responses. Communications from mitochondria to the nucleus allow cells to integrate the information on their energetic and metabolic status and align levels and spectrum of relevant bioactivities in mitochondria and nucleus, thus enabling coordinated cellular adaptation.

## Mechanisms of mitochondrial regulation of gene expression

Metabolite signaling represents one of the major modes of mitochondrial regulation of gene expression. Mitochondria produce various metabolites that can act as signaling molecules, directly or indirectly influencing gene expression.

Acetyl-CoA levels: Produced by pyruvate dehydrogenase and fatty acid oxidation, acetyl-CoA is a substrate for histone acetylation, a crucial epigenetic modification that generally promotes gene expression (59, 60).

alpha-Ketoglutarate (alpha-KG): A tricarboxylic acid (TCA) cycle intermediate, alpha-KG is a co-factor for ten-eleven translocation (TET) dioxygenases, which are involved in DNA demethylation, and Jumonji C domain-containing histone demethylases (JMJDMs), impacting chromatin structure and gene accessibility (61). This regulatory pathway is crucial for the pluripotency maintenance and self-renewal of embryonic stem cells.

NAD+/NADH Ratio: The cellular redox state, reflected by the NAD+/NADH ratio, is largely determined by mitochondrial metabolism. This ratio influences the activity of sirtuins (SIRT1, SIRT3), NAD+-dependent deacetylases that regulate gene expression by deacetylating histones and transcription factors (62).

Reactive Oxygen Species (ROS): While high levels of ROS are damaging, low, physiological levels of mitochondrial ROS can act as signaling molecules. ROS can activate various transcription factors (e.g., NF-κB, AP-1) and signaling pathways (e.g., MAPKs), leading to changes in gene expression related to stress responses, inflammation, and cell proliferation (63, 64).

Mitochondrial DNA (mtDNA) Release: Under stress conditions, mtDNA can be released into the cytoplasm, where it is recognized by innate immune sensors (e.g., cGAS-STING pathway). This triggers inflammatory responses and the upregulation of interferon-stimulated genes as well as activate the NLRP3 inflammasome (65, 66).

Mitochondrial Proteins and Peptides: Certain mitochondrial proteins or small peptides derived from them can translocate to the nucleus and directly influence gene expression. For instance, mitochondrial peptides like MOTS-c have been shown to regulate nuclear gene expression related to metabolism, reduces obesity and insulin resistance (67).

Direct Effects on Transcription Factors and Chromatin States: Mitochondrial metabolites and enzymes regulate nuclear chromatin-modifying enzymes, chromatin remodeling, and transcription regulators. Some mitochondrial proteins can interact directly with nuclear transcription factors, modulating their activity and/or localization to control differentiation, stem cells, and immune response (68). We experiencing the constant advances in our understanding of mechanisms and functional implications of direct and indirect effects of mitochondria on gene expression, both in mitochondria and the nucleus. Considering this progress together with well-documented profound regulatory effects of thyroid hormone(s) on gene expression via genomic and non-genomic mechanisms of actions, it seems reasonable to expect the emergence of novel experimental and fundamental insights into cross-talks between thyroid hormone(s) and mitochondrial pathways at the level of gene expression regulatory networks.

## Changes of thyroid hormone levels and activities during aging

Various components of endocrine systems, including the thyroid hormone axis, undergo significant changes during aging. These aging-associated changes affect the thyroid gland, levels of thyrotropin (Thyroid Stimulating Hormone), levels of total and free T4 and T3, as well as thyroid hormone metabolite reverse T3.

### Changes of thyroid-stimulating hormone levels during aging

One of the most consistently reported changes with aging is an increase in serum Thyroid-Stimulating Hormone (TSH) levels (69). Numerous studies, including large population-based surveys like NHANES III, demonstrate a progressive shift in the serum TSH distribution curve towards higher values with advancing age, even in individuals without overt thyroid disease or antibodies (70, 71). Longitudinal data also suggest that TSH generally increases over time in the same subject, particularly in older individuals (70). The traditional TSH reference range (typically 0.4-4.0 mU/L) is largely derived from younger adult populations, and there are strong arguments for the establishment and utilization of age-specific TSH reference intervals. For instance, studies have shown the upper limit of normal TSH can be significantly higher (e.g., up to 7.5 mU/L for those over 80 years) compared to younger adults (70, 72). Using age-specific reference intervals has been shown to significantly decrease the diagnosis of subclinical and overt hypothyroidism in older adults (72). The increased TSH levels in the elderly raise questions about the necessity and potential harm of treating mild TSH elevations in asymptomatic older adults. Some studies suggest that slightly higher TSH levels in the very old (e.g., >85 years) may even be associated with better survival or no adverse outcomes, advocating for a more conservative approach to treatment (70, 73). Intriguingly, he TSH population shifts to higher concentrations with age appear to be a continuum that extends even to people with exceptional longevity (74). The inverse correlation between TSH and FT4 in human populations suggests that changes in negative feedback may contribute to exceptional longevity. The TSH frequency distribution curve of centenarians was relatively similar in shape to controls but shifted significantly to higher TSH, including TSH concentration at peak frequency (74). The TSH distribution curve of the NHANES control group was superimposable to and not significantly different from the Ashkenazi controls. FT4 was similar in centenarians and Ashkenazi controls, and there was a significant inverse correlation between FT4 and TSH in both groups (74). Consistent with these observations, it has been reported that low thyroid activity in humans constitutes a heritable phenotype that contributes to exceptional familial longevity previously observed in the Leiden Longevity Study (73, 75).

### Changes of thyroid hormone (T4 and T3) levels during aging

Changes in the thyroid hormones Thyroxine (T4) and Triiodothyronine (T3) are also observed with aging, although they present a more nuanced picture than TSH.

Serum total and free T4 (FT4) concentrations generally remain relatively unchanged or show only minor changes with advancing age in healthy individuals (70, 5). While some studies have reported a slight increase in FT4 over time, this is not a universal finding, and some suggest FT4 levels show a moderate trend with age (72, 76).

A clear, age-dependent decline in serum total and free T3 (FT3) levels is widely reported (77, 78). T3 is considered the more metabolically active thyroid hormone, predominantly produced in peripheral tissues from T4 conversion. Mechanistically, FT3 decline is often linked to a decreased peripheral metabolism of iodothyronine, possibly due to reduced activity of type I deiodinase (D1) in the liver, which is crucial for both T3 production and reverse T3 (rT3) clearance (78).

Serum concentrations of the T3 metabolite reverse T3 (rT3) tend to increase with age (78). This, coupled with the decline in T3, further supports the idea of altered peripheral deiodination and may indicate a shift towards a more catabolic state in some older individuals (78). Experimental evidence indicate that rT3 is active TH metabolite interacts with αvβ3 integrin receptor and restores enzyme activities in the hippocampus of hypothyroid developing rats (9). Molecular docking analysis confirmed rT3 interaction with αvβ3 integrin receptors, thus nongenomically activating signaling pathways (PKA, CaMKII, p38MAPK) that restored gamma-glutamate transferase activity (9).

### Potential clinical relevance of FT3 and FT4 changes

The relationship between FT3, FT4, and health outcomes in the elderly is still an area of active research (79). Some studies suggest that higher FT4 within the normal range in elderly men is associated with lower physical function, while isolated low T3 might be linked to better physical performance and lean body mass. Importantly, some research indicates that higher percentiles of the FT4 reference range and lower percentiles of the TSH reference range in the elderly population are associated with higher rates of death from any cause, including cardiovascular diseases (80). Consistently, it has been observed that a lower family mortality history score (reflecting less mortality) of the parents of nonagenarian siblings was associated with higher serum TSH levels (P = 0.005) and lower free T4 levels (P = 0.002) as well as lower free T(3) levels (P = 0.034) in the nonagenarian siblings (75). Relative prevalence of FT4 observed during aging may facilitate its actions via thyrointegrin receptors, thus increasing the likelihood of malignancy-promoting activity of T4 in aging tissues.

### Changes of thyroid gland morphology and function during aging

Beyond hormone levels, the thyroid gland itself undergoes significant age-related alterations.

*Changes of Size and Location:* The thyroid gland may shrink and shift lower in the neck with age.

*Increased Prevalence of Nodules and Autoantibodies:* There is an age-dependent increase in the prevalence of thyroid nodules and positive anti-thyroperoxidase (anti-TPO) and anti-thyroglobulin (anti-Tg) antibodies, particularly in females over 60 years of age (81–83). Thyroid nodules are found in up to 50% of the population over the age of 60, and prevalence increases significantly with advancing age (82, 83). This contributes to the increased prevalence of (subclinical) thyroid disease in the elderly.

*Subclinical Hypothyroidism:* Subclinical hypothyroidism, characterized by elevated TSH and normal free thyroid hormones, is significantly more common in older adults, with prevalence estimates ranging from 5% to 20% in this age group, affecting up to 15% of those aged 65 and older when non-age-specific reference ranges are used (70, 84, 85). Its clinical significance in the elderly is still debated, with some research suggesting it may not always be associated with adverse outcomes (70, 84).

Therefore, thyroid hormone levels and activities change significantly with aging. These changes include an increase in TSH, a decline in T3, increase in rT3, and relatively stable T4 levels, alongside an increased prevalence of thyroid autoantibodies and nodules. Understanding these age-related physiological adaptations is crucial for accurate diagnosis and appropriate management of thyroid conditions in the elderly, preventing both over-treatment and under-treatment. The ongoing research into age-specific reference ranges and the clinical implications of these hormonal shifts shall continue to refine our approach to thyroid health in an aging population. This effort should include in-depth *in vivo* studies of largely neglected thyroid hormone metabolites such as rT3 and Tetrac, with the particular attention to the apparently antagonistic Tetrac and T4 actions via cell membrane thyrointegrin receptors.

THs regulatory system undergo dramatic changes during aging (**Table 3**). Specifically, functions of THs axis significant changes with advancing age:

- TSH Levels: Serum Thyroid-Stimulating Hormone (TSH) levels progressively increase in the elderly. This shift supports the need for development of healthy aging-specific TSH references and the use of age-specific TSH reference intervals.
- T4 and T3 Levels: Serum Free T4 (FT4) concentrations generally remain relatively unchanged or show only minor changes, while total and free T3 levels show a clear, age-dependent decline.
- Longevity Link: A consistent finding in longevity studies is that lower thyroid activity (higher TSH, lower FT4, and T3) is associated with exceptional familial longevity. The relative stability of FT4 in aging tissues, coupled with the decline in T3, is hypothesized to increase the likelihood of FT4-mediated, malignancy-promoting activities via the thyrointegrin receptor.

**Table 3:** Pathophysiological links to aging and cancer.

Area of pathophysiology	Key finding/Observation	Role of thyroid hormone axis	Therapeutic implication
Aging (Systemic)	Lower systemic thyroid activity (higher TSH, lower T3) associated with aging.	Age-dependent T3 decline is observed. T4 stability may increase non-genomic T4-Integrin signaling.	Homeostasis: Healthy aging-appropriate TSH and TH reference ranges are needed.
Malignant Transformation	Cancer stem cells (CSCs) exhibit mitochondrial phenotypes optimized for survival.	T4 binding to αVβ3 integrin promotes cell survival, stemness, and proliferation.	Targeting: Development of Nanotetrac-like molecules to block T4’s pro-malignancy signaling pathway.
Immune Evasion (Cancer)	Cancer cells transfer defective mitochondria containing mutant-mtDNA to immune cells (TILs).	THs/Integrin signaling may drive mitochondrial hijacking mechanisms.	Immunotherapy: Selective targeting mitochondrial transfer could restore T-cell function and anti-tumor immunity.

## Conclusion

Review of the effects of THs and their metabolites on classical and non-canonical mitochondrial structure and functions in various pathophysiological conditions, including aging, stemness, and cancer, indicates that nongenomic mode of actions of THs on mitochondria appear affected during aging and alterations of nongenomic pathways of THs actions may contributed to development of cancer and other aging-associated disorders.

**Figure 1:**
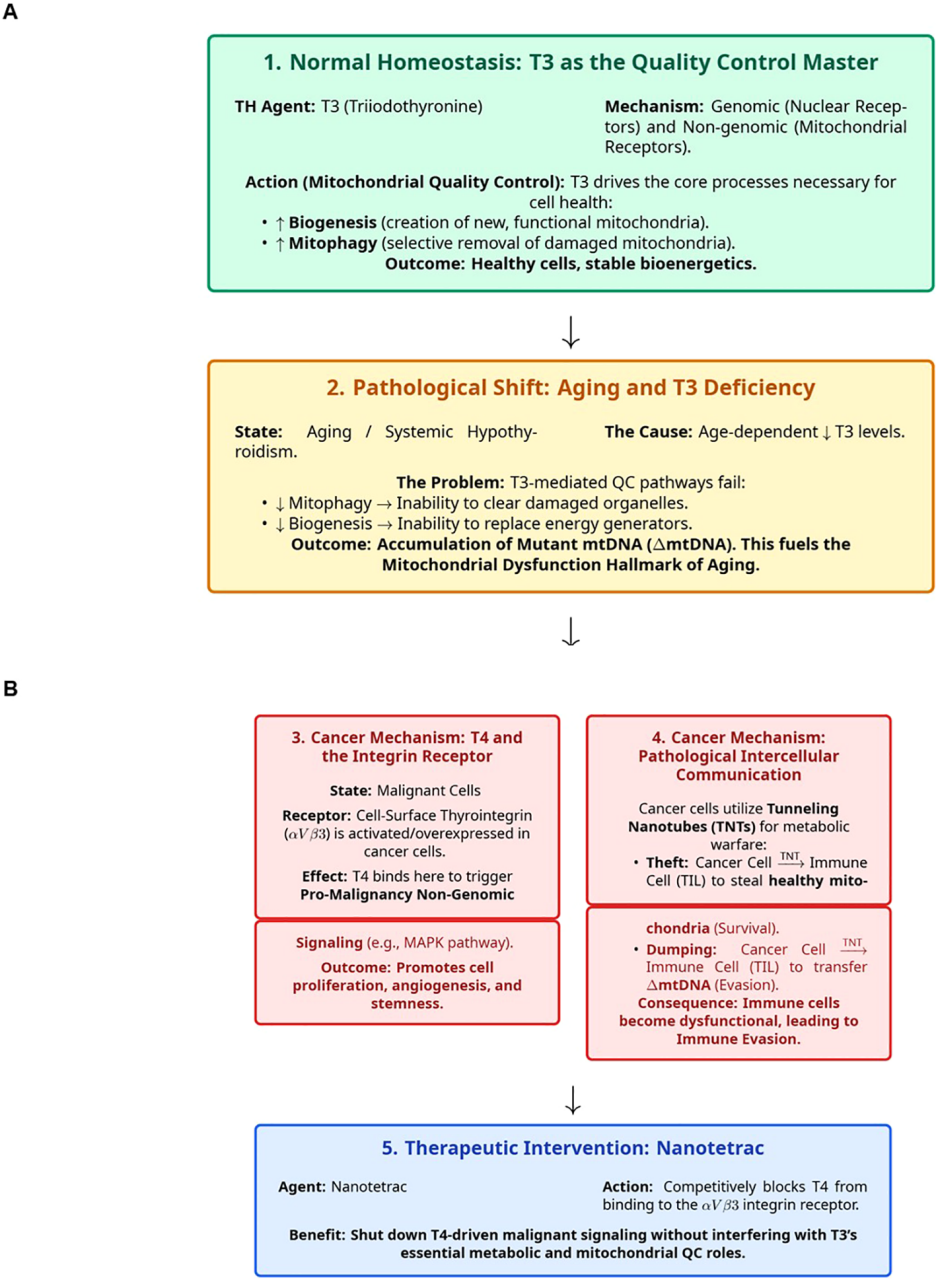
Graphical summary of regulatory impacts of thyroid hormones (TH) on mitochondria and the mitochondrial dysfunction axis in aging and cancer. **(A)** This panel summarizes actions of THs in physiological conditions designed to maintain organisms’ and individual tissues’ homeostasis, support healthy cells, and ensure the stable and balanced bioenergetics (top box 1). The pathological shift of the THs system functions during aging is illustrated in the bottom box 2. **(B)** This panel illustrates two cancer driving pathological mechanisms exerted by non-genomic actions of T4 via thyrointegrin receptors (Box 3) and pathology-enabling intercellular communications which are based on cancer cells abilities to utilize the Tunneling Nanotubes (TNT) malignancy-promoting metabolic warfare. The bottom box 5 outlines the therapeutic potential of the Nanotetrac-based molecules to shut-down T4-driven malignancy-promoting signaling.

The findings presented in this paper fundamentally reframe the role of thyroid hormones, moving beyond their classical metabolic functions to establish a critical axis linking thyroid hormone signaling, mitochondrial quality control, and the pathologies of aging and pathogenesis of malignancy (**Figure 1**). We have reported the evidence that conclusively demonstrated that T3 is a primary top-level hormonal regulator of mitochondrial homeostasis, orchestrating the essential balance of biogenesis and mitophagy that defines cellular health. The age-dependent decline in T3 signaling thus emerges as a key mechanistic driver for the accumulation of mutant mtDNA, a principal hallmark of aging (86).

Detailed estimates of the prevalence of adult mitochondrial diseases caused by mutations in mtDNA is estimated at 9.6 cases per 100,000 individuals and the prevalence of mitochondrial diseases caused by mutations in nDNA is estimated at 2.9 cases per 100,000 individuals (87, 88). Results of the analytical inquiries reported in this contribution strongly argue that mitochondrial dysfunctions caused by the aberrant actions of THs system may cause the mitochondrial pathologies during aging, cancer, and multiple other age-associated pathological conditions. Consistent with this concept, the recent study investigating the effect of the mitophagy inducer urolithin A (UA) on age-related immune decline provided clinical evidence that short-term UA supplementation modulates human immune cell composition and function, supporting its potential to counteract age-related immune decline and inflammaging (89).

Furthermore, we highlighted research that illuminated how malignant cells hijack and corrupt this regulatory axis for their own survival. Cancer subverts the system through two distinct but complementary pathways. First, it overexpresses the cell-surface integrin αVβ3, repurposing T4 as a potent, non-genomic driver of proliferation and angiogenesis. Second, it engages in a form of metabolic warfare, utilizing Tunneling Nanotubes (TNTs) to actively sabotage the tumor microenvironment by stealing healthy mitochondria from normal cells, and dumping damaged mutant mtDNA into responding to malignant growth immune cells, thereby rendering them non-functional and ensuring its own survival and evasion.

The critical insight from this work is the identification of the αVβ3 integrin as a highly specific, high-value potential therapeutic target. By decoupling the pathological, malignancy-promoting T4-driven pro-cancer signals from the essential, T3-driven physiological functions, we highlighted a new therapeutic window. Agents such as Nanotetrac, which selectively block T4 binding at the integrin, represent one of the promising strategies. This approach carries the potential to neutralize a key driver of malignancy and immune evasion without inducing the systemic metabolic disruption of traditional thyroid-targeting therapies, offering a tailored and potent new weapon in the fight against cancer.
